# Dynamic Changes of Microbial Communities and Chemical Compounds During the Dry Processing of *Coffea arabica*

**DOI:** 10.1002/fsn3.70178

**Published:** 2025-04-24

**Authors:** Xiaojing Shen, Qi Wang, Biao Yuan, Zhiheng Yin, Muzi Li, Runxin Shi, Kunyi Liu, Wenjuan Yuan

**Affiliations:** ^1^ College of Science & College of Food Science and Technology Yunnan Agricultural University Kunming Yunnan China; ^2^ School of Wuliangye Technology and Food Engineering Yibin Vocational and Technical College Yibin China

**Keywords:** bacteria, differentially changed compounds, fungi, high‐throughput sequencing technology, HPLC‐MS/MS

## Abstract

Coffee is a globally popular beverage and a significant agricultural economic crop in planted countries and regions. To understand the influence of primary processing on coffee, the microbial communities and chemical compounds were analyzed using high‐throughput sequencing and HPLC‐MS/MS metabolomics to confirm the dynamic changes of them during the dry processing of *Coffea arabica* from Yunnan, China. The results showed that *Tatumella*, *Klebsiella*, *Gluconobacter*, *Brevundimonas*, and *Staphylococcus* at the bacterial general level and *Candida*, *Lachancea*, *Aschersonia*, *Cercospora*, and *Pichia* at the fungi general level were the predominant microorganisms in the dry processing. During the dry process, *Tatumella*, *Gluconobacter*, and *Candida* showed a trend of increasing firstly and decreasing subsequently, while *Klebsiella* and *Lachancea* reached the highest at the end of processing. In contrast, *Hannaella* decreased gradually. Meanwhile, 1551 chemical compounds coming from 15 superclasses were detected. Furthermore, 129 differentially changed compounds (DCCs) including 91 upregulated DCCs with VIP > 1.0, *p* < 0.05, and FC > 1.5 and 38 downregulated DCCs with VIP > 1.0, *p* < 0.05, and FC < 0.67 were determined in finished samples versus raw materials. Among them, cis‐5‐caffeoylquinic acid and sesamose were very significantly DCCs. Therefore, this study can provide useful information for understanding the dry processing of coffee beans and its impact on coffee's quality properties.

## Graphical abstract

*Tatumella*, *Klebsiella*, *Gluconobacter*, *Brevundimonas*, and *Staphylococcus* at the bacterial general level and *Candida*, *Lachancea*, *Aschersonia*, *Cercospora*, and *Pichia* at the fungi general level were the predominant microorganisms during the dry processing of *Coffea arabica* . Meanwhile, 129 differentially changed compounds (DCCs) including 91 upregulated DCCs with VIP > 1.0, *p* < 0.05, and FC > 1.5, and 38 downregulated DCCs with VIP > 1.0, *p* < 0.05, and FC < 0.67, were determined in finished samples versus raw materials.

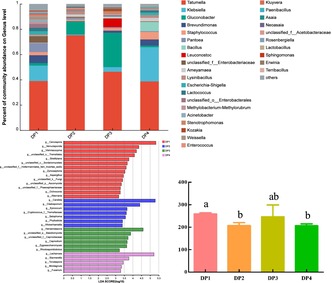

## 1 Introduction

Coffee, as one of three beverages globally, not only has an attractive flavor and taste but also has multiple health functions for humans, such as cardioprotection and longevity, improving mental health, antioxidant activity, anti‐diabetic effect, cancer chemoprevention (Freitas, Borges, Castro, et al. 2024; Freitas, Borges, Vidigal, et al. 2024; Ismail et al. 2021), etc. Washed, dry, and semidry processing are three traditional postharvest primary processing methods for obtaining green coffee beans, which are crucial influences on the composition and flavor of coffee in coffee production industrialization (Freitas, Borges, Castro, et al. 2024; Freitas, Borges, Vidigal, et al. 2024). Because the coffee processing method can influence endogenous and exogenous factors, such as the metabolism of the coffee fruit, microbial ecology, and fermentation conditions (Ferreira et al. 2023).

The primary processing method brings the fermentation of coffee fruits by different microorganisms, which play a crucial role in producing enzymes to degrade pectin and metabolites to change chemical compounds in coffee fermentation (Shen et al. 2023a). Furthermore, the microbial community was a dynamic change process during primary processing (Shen et al. 2025). The dry processing is also called natural processing, in which whole mature coffee fruits are dried under the sun for about 21 days on cement platform terraces; then, the dried coffee fruits were removed until the moisture content is about 12% (Elhalis et al. 2023; Lee et al. 2015). Dry processing is beneficial to the fruity, floral, and sweet aromas of coffee beverages (Zhai et al. 2024). During dry processing, coffee undergoes spontaneous fermentation and the pulp and mucilage are broken by the natural microbial fermentation and enzymatic actions under the coffee cherries intact (Shen et al. 2025; Lee et al. 2015). During the dry processing, main microorganisms including *Erwinia*, *Enterobacter*, *Klebsiella*, *Tatumella*, *Pseudomonas*, *Proteus*, *Acinetobacter*, *Bacillus*, *Lactobacillus*, and *Leuconostoc* genera from bacteria, *Debaryomyces*, *Pichia*, and *Arxula* genera from Yeast, *Cladosporium*, *Fusarium*, *Pestalotia*, *Paecelomyces*, and *Aspergillus* genera from filamentous fungi were found (Elhalis et al. 2023). However, microorganisms are different in different primary processing methods. For example, *Achromobacter*, *Tatumella*, *Weissella*, *Streptococcus*, and *Trichocoleus* for bacteria and *Cystofilobasidium*, *Hanseniaspora*, *Lachancea*, *Wickerhamomyces*, and *Aspergillus* for fungi were the top predominant microorganisms in the wet processing (Shen et al. 2023b). *Tatumella*, *Staphylococcus*, *Klebsiella*, *Brevundimonas*, and *Gluconobacter* for bacteria and *Candida*, *Hannaella*, *Hanseniaspora*, *Pichia*, and *Lachancea* for fungi were the most abundant genera in the semidry processing (Shen et al. 2024).

*Coffea arabica* from Yunnan shows a coverage sensory profile and a high quality (Ma et al. 2022). Yunnan province is an ideal coffee‐growing region, which accounts for over 99% of China's coffee plantation area and mainly grows *C. arabica* (Ma et al. 2022). In 2022, the total coffee cultivation area in Yunnan was 86,667 hm2, and approximately 110,000 tons of green coffee beans were produced. The data from USDA showed production of *C. arabica* from China reached 1700 thousand bags accounting for 1.75% of the world's total *C. arabica* production and 0.99% of the world's total coffee production, respectively, in December 2023/2024. To further understand the change in microorganisms and chemical compounds during the dry processing, the microbial community structure and chemical compounds of *C. arabica* from Yunnan province were analyzed in this paper.

## 2 Materials and Methods

### 2.1 Plant Material and Reagents

Mature *C. arabica* cherries were collected in December 2023 from Pu‐er, China. Methyl alcohol, acetonitrile, and propyl alcohol were high‐performance liquid chromatography (HPLC) grade and purchased from Fisher Co. Ltd. (Shanghai, China).

### 2.2 Sample Preparation

After harvesting, mature *C. arabica* cherries were processed using the dry processing method, which consisted of flotation and drying (Lee et al. 2015). During the dry processing, four coffee bean samples were obtained at 0 days (DP1), 5 days (DP2), 10 days (DP3), and 15 days (DP4) (*n* = 3), respectively.

### 2.3 Analysis of Microbial Communities During Dry Processing

The microbial communities of four coffee samples during the dry processing were analyzed using high‐throughput sequencing technology. They were carried out by Majorbio Bio‐Pharm Technology Co. Ltd. (Shanghai, China) referencing the method of Shen et al. (Shen et al. 2023b). For bacteria, the hypervariable region V_5_‐V_7_ of the 16S rRNA gene was amplified with forward primer 799F and reverse primer 1193R. Meanwhile, the ITS1 region used ITS1F and ITS2R primers for fungi. Finally, the raw sequencing reads of bacterial 16S rRNA and fungal ITS1 were deposited into the NCBI Sequence Read Archive database (Liu et al. 2022; Zhao et al. 2022), and the accession numbers were PRJNA1089976 and PRJNA1089988, respectively.

### 2.4 Analysis of Chemical Compounds During Dry Processing

Four coffee extracts during the dry processing using 0.4 mL of an 80% methanol solution were centrifuged at 13,000 g for 15 min at 4°C to obtain the supernatant for HPLC‐MS/MS analysis, which was carried out using the chromatographic separation performed using an HSS T3 C_18_ column (2.1 × 100 mm, 1.8 μm; Waters Corporation, Milford, MA, USA) by referencing the process of Shen et al. (2023b). The mobile phase consisted of a blend of (A) 0.1% formic acid in water: acetonitrile (95:5, v/v) and (B) 0.1% formic acid in acetonitrile: isopropanol: water (47.5:47.5:5, v/v). The gradient elution proceeded as follows under a flow rate of 0.4 mL/min: 0%–5% B for 0–0.1 min; 5%–25% B for 0.1–2 min; 25%–100% B for 2–9 min; 100% B for 9–13 min; and 100%–0% B for 13–13.1 min; then 0% B for 13.1–16 min for system equilibration. The optimal conditions for the mass spectrum were as follows: heater temperature at 400°C; sheath gas flow rate at 40 arb; aux gas flow rate at 10 arb; ion‐spray voltage floating (ISVF) at −2800 V in negative mode and 3500 V in positive mode; and normalized collision energy set at 20–40–60 V for MS/MS. The detection range covered a mass range of 70–1050 m/z. Quality control (QC) samples were prepared by combining equal volumes of all samples.

### 2.5 Statistical Analysis

Each coffee sample was replicated three times. Variable importance in projection (VIP) analysis ranked the overall contribution of each variable to the OPLS‐DA model. The differentially changed compounds (DCCs) were determined with VIP > 1.0, *p* < 0.05, and fold change (FC) > 1.5 or < 0.67.

## 3 Results

### 3.1 The Analysis Results of Microbial Communities During Dry Processing

**FIGURE 1:**
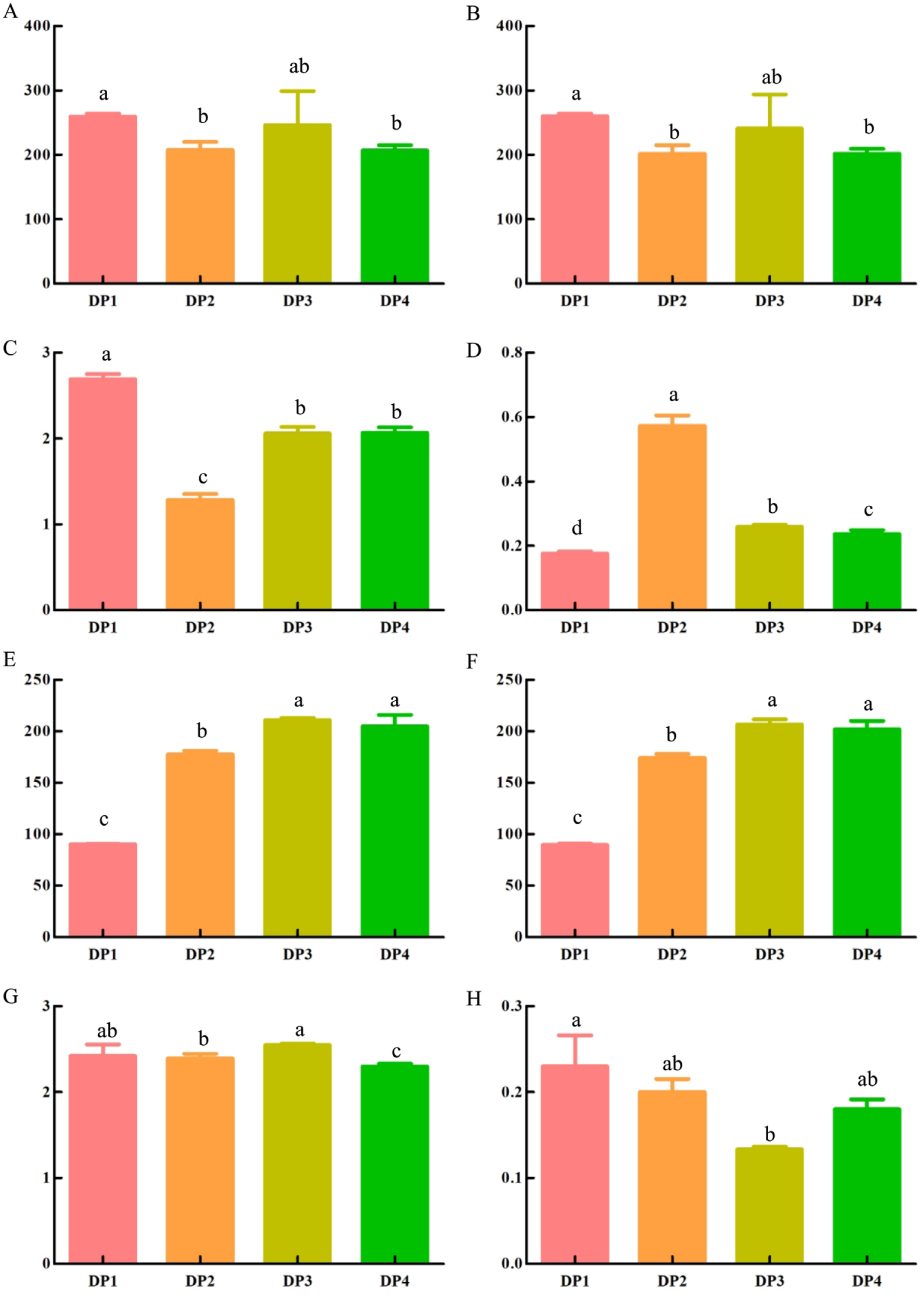
The analysis results of microbial alpha diversity during the dry processing in *C. arabica* from Yunnan province, China. Different lowercase superscripts indicated significantly different among comparisons (*p* < 0.05). (A–D: Ace, Chao, Shannon, and Simpson in bacteria, respectively; E‐H: Ace, Chao, Shannon, and Simpson in fungi, respectively.)

During the dry processing of *C. arabica* , a total of 740,144 bacterial sequences and 1,219,337 fungal sequences were determined, respectively. The coverage of coffee samples in each group was higher than 0.99, which indicated that the microbial diversity of these coffee samples was comprehensive and accurate (Wang et al. 2023). Figure 1 showed the analysis result of microbial alpha diversity during the dry processing, which was used to reflect the complexity of microorganisms within the coffee sample (Wang et al. 2023). At the operational taxonomic unit (OUT) level, the *p*‐values of the ace index were 0.28 and 0.024 in bacteria and fungi, respectively (Figure 1A,E). The richness and diversity of species are reflected by Chao and Shannon indices (Wang et al. 2023). The *p*‐values of the Chao index were 0.26 in bacteria and 0.024 in fungi. Among bacteria, DP1 had the highest Chao and Shannon indices (Figure 1B,C). Among fungi, DP3 showed the highest Chao and Shannon indices (Figure 1F,G), which indicated DP1 and DP3 had the highest richness and diversity of species in bacteria and fungi, respectively. At the same time, DP2 and DP1 showed the highest Simpson indices for bacteria and fungi, respectively (Figure 1D,H), which means they showed the highest diversity (Wang et al. 2020).

**FIGURE 2:**
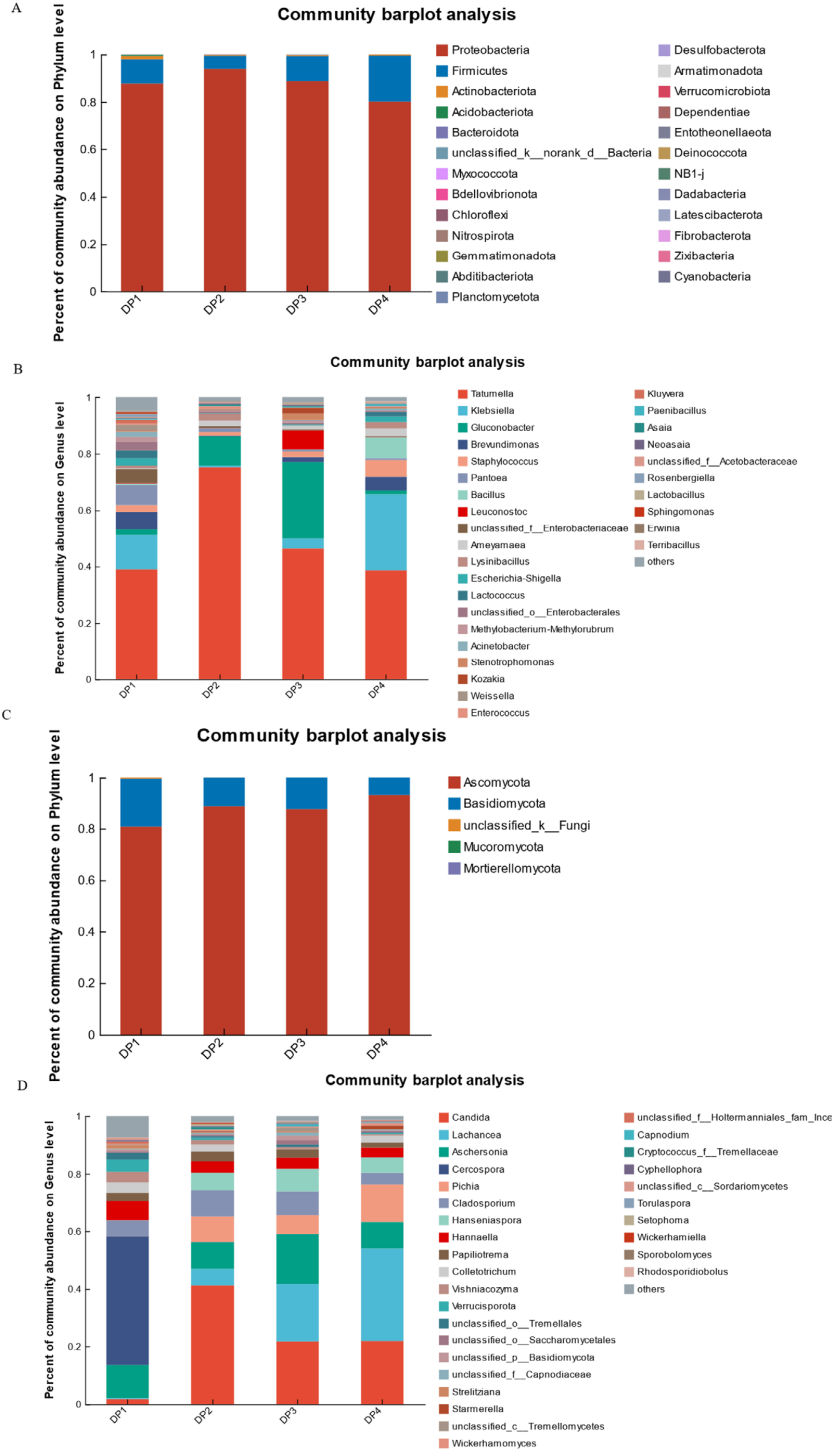
Microbial community structure during the dry processing in *C. arabica* from Yunnan province, China. (A: The percent of community abundance on the phylum level of bacteria; B: The percent of community abundance on the genus level of bacteria; C: The percent of community abundance on the phylum level of fungi; D: The percent of community abundance on genus level of fungi).

The bacteria during the dry processing were classified into 25 phyla, as shown in Figure 2A. These phyla included *Proteobacteria*, *Firmicutes*, *Actinobacteriota*, *Acidobacteriota*, *Bacteroidota*, *Myxococcota*, *Bdellovibrionota*, *Chloroflexi*, *Nitrospirota*, *Gemmatimonadota*, *Abditibacteriota*, *Planctomycetota*, *Desulfobacterota*, etc. Among them, the dominant phyla were *Proteobacteria* (comprising 80.16%–93.91% of the community abundance on phyla level), *Firmicutes* (5.49%–19.31%), and *Actinobacteriota* (0.02%–1.44%). Notably, the relative percent of *Proteobacteria* in all the samples was more than 80.00%. The relative percent of *Proteobacteria* community abundance initially increased from 87.77% in DP1 to 93.91% in DP2, then decreased to 80.16% in DP4. Compared with *Proteobacteria*, *Firmicutes* exhibited significant opposite changes, which decreased from 10.13% in DP1 to 5.49% in DP2, then increased to 19.31% in DP4.

31 bacterial genera were confirmed during the dry processing, as shown in Figure 2B. They included *Tatumella*, *Klebsiella*, *Gluconobacter, Brevundimonas*, *Staphylococcus*, *Pantoea*, *Bacillus*, *Leuconostoc*, *Ameyamaea*, *Lysinibacillus*, *Lactococcus*, etc. At the beginning of the dry processing, *Tatumella* and *Klebsiella* were the dominant bacteria at the genus level, with 39.01% and 12.17% in DP1, respectively. Then, *Tatumella* significantly increased to 75.21% in DP2, while *Klebsiella* declined to its lowest abundance of 0.58. In DP3, *Tatumella* significantly decreased to 46.36%. *Klebsiella* increased to 3.60% in DP3 and reached the highest 27.09% in DP4. In contrast, *Gluconobacter* showed a similar pattern to that of *Tatumella*, increasing during the first 10 days and then decreasing.

**FIGURE 3:**
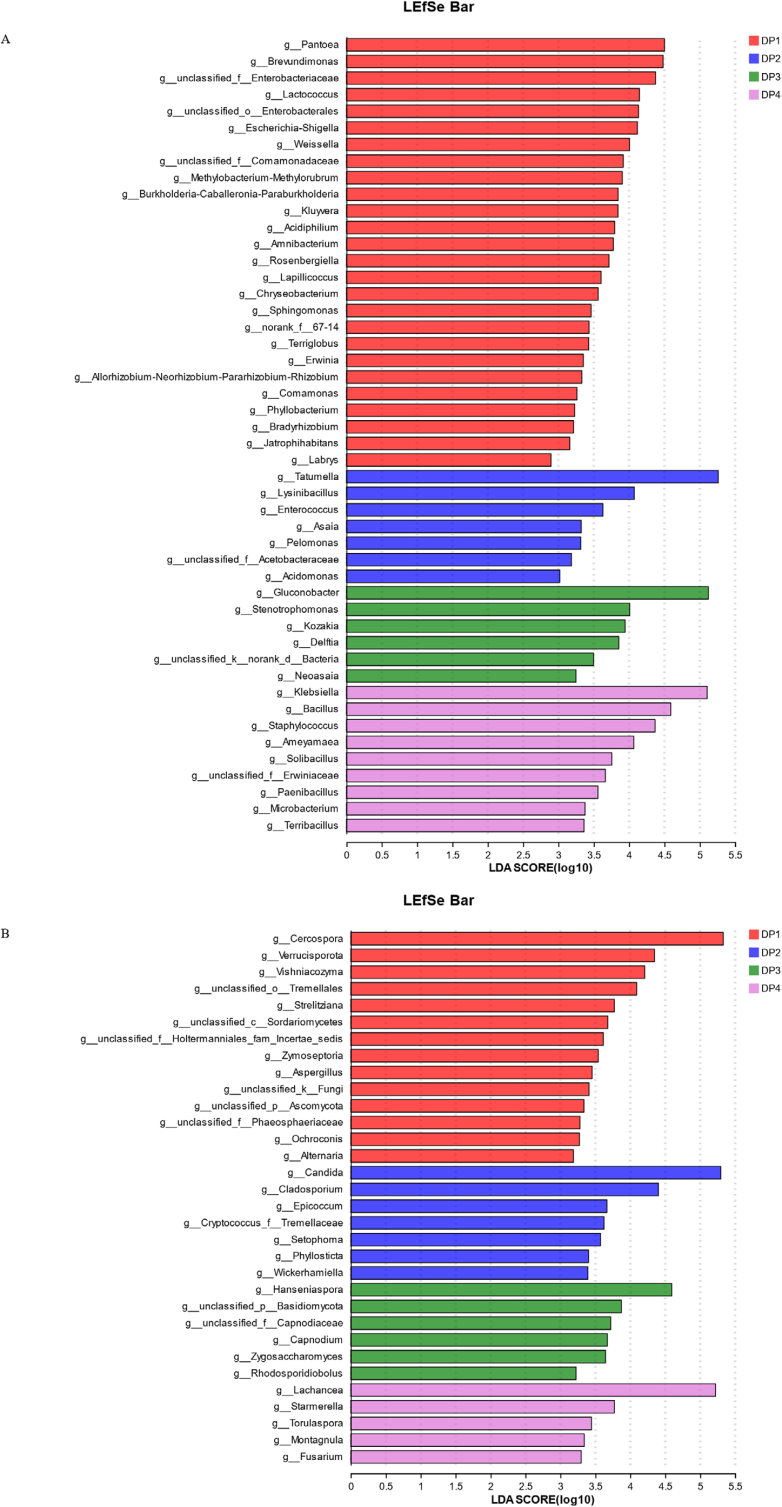
LEfSe bar during dry processing in *C. arabica* from Yunnan province, China. LDA score of bacteria (A); LDA score of fungi (B).

Linear discriminant analysis effect size (LEfSe) is an efficient way to select biomarkers by analyzing characterized statistical differences among biological groups. Linear discriminant analysis (LDA) was used to evaluate the influence of biomarkers on significantly different groups based on LDA scores (Chang et al. 2022). The LEfSe results on the genus levels are shown in Figure 3. For bacteria (Figure 3A), among them, 26 genus‐level bacteria, such as *Pantoea* (LDA score = 4.50, *p* = 0.024), *Brevundimonas* (LDA score = 4.48, *p* = 0.019), *Lactococcus* (LDA score = 4.15, *p* = 0.016), and *Weissella* (LDA score = 4.01, *p* = 0.021), were significantly higher in DP1. 7 geneus levels, such as *Tatumella* (LDA score = 5.26, *p* = 0.025), *Lysinibacillus* (LDA score = 4.07, *p* = 0.024), *Enterococcus* (LDA score = 3.63, *p* = 0.016), *Asaia* (LDA score = 3.32, *p* = 0.022), *Pelomonas* (LDA score = 3.32, *p* = 0.031), and *Acidomonas* (LDA score = 3.02, *p* = 0.048), were significantly higher in DP2. 6 genus‐level bacteria, such as *Gluconobacter* (LDA score = 5.12, *p* = 0.016), *Strenotrophomonas* (LDA score = 4.01, *p* = 0.017), *Kozakia* (LDA score = 3.94, *p* = 0.041), *Delftia* (LDA score = 3.85, *p* = 0.025), and *Neoasaia* (LDA score = 3.25, *p* = 0.036), were significantly higher in DP3. 9 genus‐level bacteria, such as *Klebsiella* (LDA score = 5.10, *p* = 0.016), Bacillus (LDA score = 4.59, *p* = 0.017), *Staphylococcus* (LDA score = 4.37, *p* = 0.023), *Ameyamaea* (LDA score = 4.07, *p* = 0.033), and *Solibacillus* (LDA score = 3.76, *p* = 0.020), were significantly higher in DP4.

The fungi in four different coffee samples had lower diversity than bacteria on the phyla level, which could be classified into 5 phyla as shown in Figure 2C. These phyla included *Ascomycota*, *Basidiomycota*, *Mucoromycota*, *Mortierellomycota*, and unclassified fungi. The dominant phyla were *Ascomycota* (comprising 80.81%–93.09%) and *Basidiomycota* (6.90%–18.75%) during the dry processing. Notably, the relative abundance of *Ascomycota* in different samples was consistently higher than 80.00%. The relative abundance of *Ascomycota* first increased, reaching a maximum of 93.09% in DP4. However, the relative abundance of *Basidiomycota* initially decreased, reaching a lowest of 6.90% in DP4.

Furthermore, these fungi were confirmed to belong to 31 genera, as shown in Figure 2D. They included *Candida*, *Lachancea*, *Aschersonia*, *Cercospora*, *Pichia*, *Cladosporium*, *Hanseniaspora*, *Hannaella*, *Papiliotrema*, *Colletotrichum*, *Vishniacozyma*, *Verrucisporota*, etc. The predominant genera were *Candida*, *Lachancea*, *Aschersonia*, *Cercospora*, *Pichia*, *Cladosporium*, *Hanseniaspora*, and *Hannaella*. The percent of community abundance of Candida on the genus level ranged from 1.81% to 41.26%, with the maximum value observed in DP2 and the minimum value in DP1. On the other hand, the value of *Lachancea* increased gradually during dry proceeding, with the maximum value of 32.17% in DP4 and the minimum value of 0.26% in DP1. In contrast, *Hannaella* decreased gradually during dry proceeding, with a maximum value of 6.70% in DP1 and the minimum value of 3.41% in DP4. *Aschersonia* showed a maximum of 17.20% in DP3 and a minimum of 9.17% in DP4. *Cercospora* was only found in DP1, reaching 44.70%, while *Hanseniaspora* was only 0.08%. *Pichia* was 0.04% in DP1 and increased to 13.13% in DP4. *Cladosporium* in DP2 and DP3 was richer than others, with 9.25% and 8.37%, respectively.

For fugue (Figure 3B), among them, 14 genus levels, such as *Cercospora* (LDA score = 5.33, *p* = 0.047), *Verrucisporota* (LDA score = 4.35, *p* = 0.020), *Vishniacozyma* (LDA score = 4.21, *p* = 0.016), *unclassified‐o‐Tremellales* (LDA score = 4.09, *p* = 0.019), and *Aspergillus* (LDA score = 3.45, *p* = 0.042), were significantly higher in DP1. 7 genus‐level fungi, such as *Candida* (LDA score = 5.30, *p* = 0.025), *Cladosporium* (LDA score = 4.40, *p* = 0.030), *Epicoccum* (LDA score = 3.67, *p* = 0.039), *Setophoma* (LDA score = 3.57, *p* = 0.022), and *Phyllosticta* (LDA score = 3.40, *p* = 0.022), were significantly higher in DP2. 6 genus‐level fungi, such as *Hanseniaspora* (LDA score = 4.59, *p* = 0.022), *Capnodium* (LDA score = 3.67, *p* = 0.032), *Zygosaccharomyces* (LDA score = 3.65, *p* = 0.015), and *Rhodsporidiobolus* (LDA score = 3.22, *p* = 0.043), were significantly higher in DP3. 5 genus‐level fungi, *Lachancea* (LDA score = 5.22, *p* = 0.016), *Starmerella* (LDA score = 3.77, *p* = 0.022), *Torulaspora* (LDA score = 3.44, *p* = 0.021), *Montagnula* (LDA score = 3.34, *p* = 0.044), and *Fusarium* (LDA score = 3.30, *p* = 0.015), were significantly higher in DP4.

### 3.2 The Analysis Results of Chemical Compounds During Dry Processing

**FIGURE 4:**
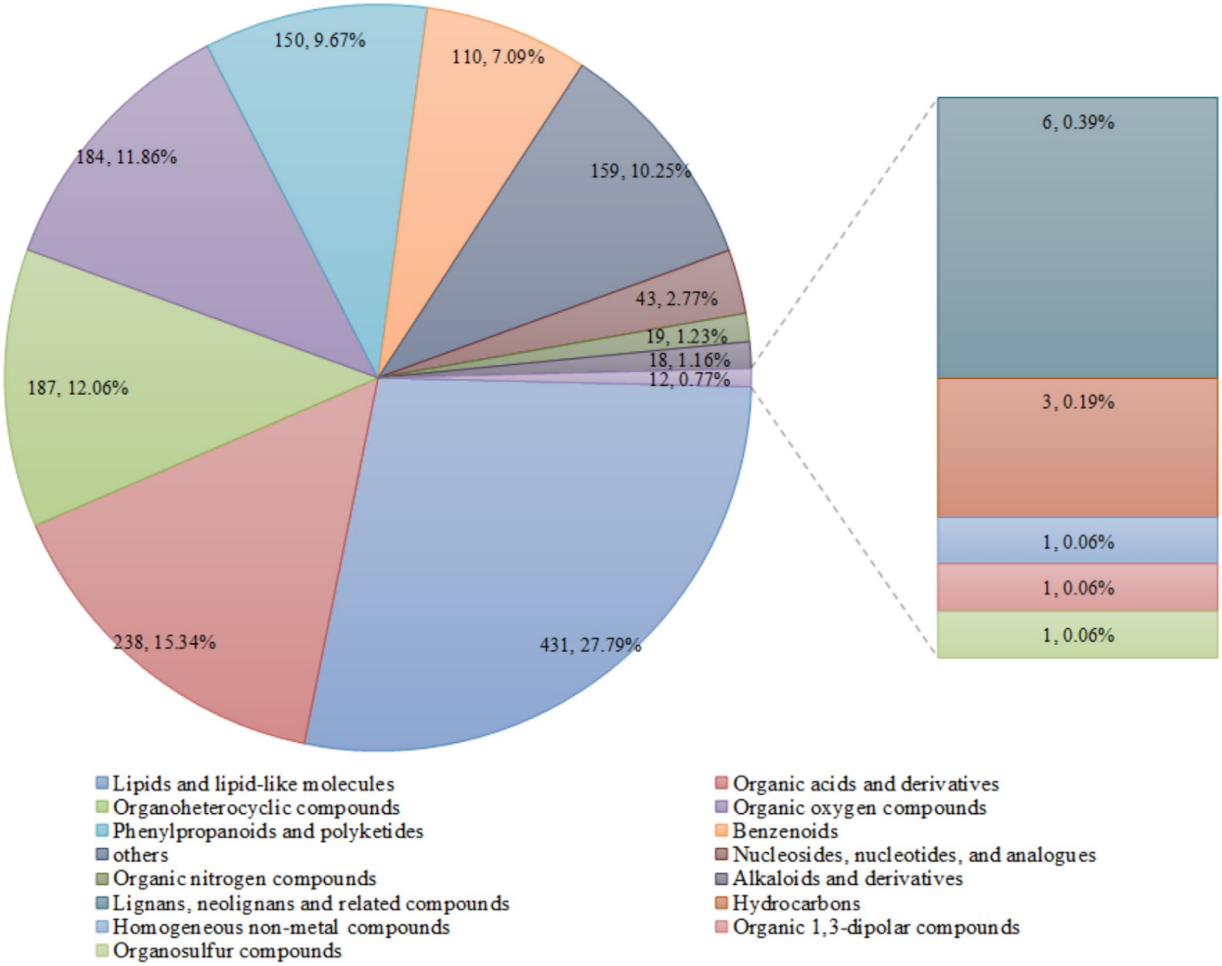
Superclasses of chemical compounds during the dry primary processing in *C. arabica* from Yunnan province, China. Different colors represent different superclasses of chemical compounds, the size represents the number of chemical compounds, and the bigger circle means the more chemical compounds.

A total of 1551 compounds classified into 15 superclasses were detected in four coffee samples during the dry processing, as shown in Figure 4. These superclasses included lipids and lipid‐like molecules (431 compounds, comprising 27.79%), organic acids and derivatives (238 compounds comprising 15.34%), organoheterocyclic compounds (187 compounds, comprising 12.06%), organic oxygen compounds (184 compounds, comprising 11.86%), phenylpropanoids and polyketides (150 compounds, comprising 9.67%), benzenoids (110 compounds, comprising 7.09%), nucleosides, nucleotides, and analogs (43 compounds, comprising 2.77%), organic nitrogen compounds (19 compounds, comprising 1.23%), alkaloids and derivatives (18 compounds, comprising 1.16%), lignans, neolignans, and related compounds (6 compounds, comprising 0.39%), hydrocarbons (3 compounds, comprising 0.19%), homogeneous non‐metal compounds (1 compound, comprising 0.064%), organic 1,3‐dipolar compounds (1 compound, comprising 0.064%), organosulfur compounds (1 compound, comprising 0.064%), and others (159 compounds, comprising 10.25%).

Of course, these compounds were further grouped into 124 classes. These classes mainly included carboxylic acids and derivatives (196 compounds), organooxygen compounds (184 compounds), fatty acyls (161 compounds), prenol lipids (118 compounds), benzene and substituted derivatives (74 compounds), steroids and steroid derivatives (66 compounds), glycerophospholipids (63 compounds), flavonoids (58 compounds), coumarins and derivatives (28 compounds), cinnamic acids and derivatives (27 compounds), indoles and derivatives (25 compounds), phenols (23 compounds), organonitrogen compounds (19 compounds), imidazopyrimidines (16 compounds), glycerolipids (15 compounds), hydroxy acids and derivatives (14 compounds), isoflavonoids (14 compounds), purine nucleosides (14 compounds), benzopyrans (12 compounds), diazines (11 compounds), lactones (10 compounds), keto acids and derivatives (10 compounds), and more.

**FIGURE 5:**
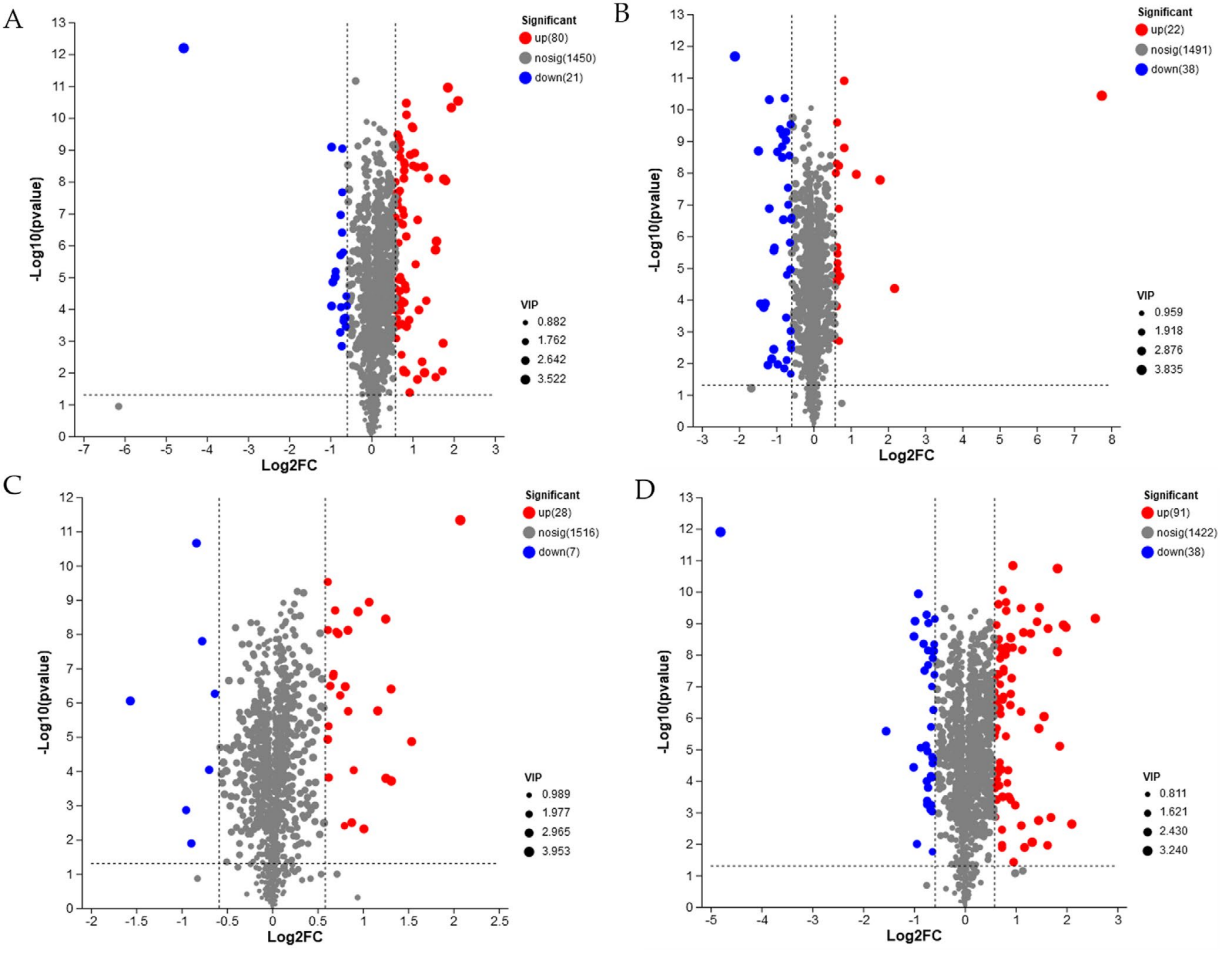
The differently changed compounds during the dry processing in *C. arabica* from Yunnan province, China. 101 DCCs between DP2 and DP1 (A), including 80 upregulated DCCs and 21 downregulated DCCs; 60 DCCs between DP3 and DP2 (B), including 22 upregulated DCCs and 38 downregulated DCCs; 35 DCCs between DP4 and DP3 (C), including 28 upregulated DCCs and 7 downregulated DCCs; 129 DCCs between DP4 and DP1 (D) including 91 upregulated DCCs and 38 downregulated DCCs.

The significant differentially changed compounds (DCCs) between DP1, DP2, DP3, and DP4 with VIP > 1.0, *p* < 0.05, and FC > 1.5 or FC < 0.67 were analyzed to gain further information about the dry processing, as shown in Figure 5. 101 DCCs were detected between DP2 and DP1 (Figure 5A). Among these DCCs, 80 DCCs were upregulated (FC > 1.5), including organic acids and derivatives (18 DCCs, e.g., Na‐p‐hydroxycoumaroyltryptophan, cysteinyl‐alanine, gluten exorphin B4, N‐eicosapentaenoyl aspartic acid, N‐carbamoylputrescine, etc.), organic oxygen compounds (16 DCCs, e.g., 2‐fucosyllactose, lichenin, cis‐5‐caffeoylquinic acid, 4‐*O*‐alpha‐D‐galactopyranuronosyl‐D‐galacturonic acid, 1‐(3,4‐dimethoxyphenyl)‐1,2‐ ethanediol 2‐O‐b‐D‐glucoside, etc.), lipids and lipid‐like molecules (15 DCCs, e.g., alfaprostolum, PA(i‐21:0/8:0), 17‐hydroxy‐3,11,20‐trioxopregn‐4‐en‐21‐yl acetate, isopropylmaleic acid, pentadeca‐7,10,13‐trienedioylcarnitine, etc.), phenylpropanoids and polyketides (11 DCCs, e.g., luteolin 7‐glucuronide, nodakenetic, 2″,6″‐diacetylorientin, biochanin A 7‐(6‐malonylgucoside), betavulgarin, etc.), organoheterocyclic compounds (7 DCCs, e.g., 4‐*O*‐(indole‐3‐acetyl)‐D‐glucopyranose, decoquinate, verbasoside, stercobilin, ivabradine, etc.), benzenoids (5 DCCs, 4‐benzyl‐methyl‐1,2,4‐thiadiazolidine‐3,5‐dione, chavicol, benzyl formate, 4R,5R,6S,‐trihydroxy‐2‐hydroxymethyl‐2‐cyclohexen‐1‐one 6‐(2‐hydroxy‐6‐ methylbenzoate), and N‐(6‐aminopyridin‐2‐yl)‐4′‐cyanobipheny‐4‐sulfonamide), alkaloids and derivatives (4 DCCs, lycorine, delimotecan, 10‐hydroxycamptothecin, and camptothecin sodium), and others (4 DCCs, (2S,3S,5S,8R,9S,10S,13S,14S,16S,17R)‐17‐Acetyloxy‐10,13‐dimethyl‐2‐morpholin‐4‐yl‐16‐(1‐prop‐2‐enylpyrrolidin‐1‐ium‐1‐yl)‐2,3,4,5,6,7,8,9,11,12,14,15,16,17‐tetradecahydro‐1H‐cyclopenta[a]phenanthren‐3‐olate, N‐benzyloxycarbonylglycine, nodakenetic, and scorodocarpine A). Among them, 8 upregulated DCCs (2‐fucosyllactose, luteolin 7‐glucuronide, lycorine, lichenin, 4‐benzyl‐2‐methyl‐1,2,4‐thiadiazolidine‐3,5‐dione, N‐(6‐aminopyridin‐2‐yl)‐4′‐cyanobiphenyl‐4‐sulfonamide, cysteinyl‐alanine, and Na‐*p*‐hydroxycoumaroyltryptophan) were important DCCs with the value of FC over 3.0. Meanwhile, 21 DCCs were downregulated (FC < 0.67), including lipids and lipid‐like molecules (6 DCCs, e.g., necatorine, abscisic alcohol, 3‐[(2R)‐2‐Hydroxy‐3‐methyl‐3‐[(phosphonooxy)methyl]butanamido]propanoylcarnitine, neuraminic acid, glycerol 3‐phosphate, etc.), phenylpropanoids and polyketides (4 DCCs, (+)‐taxifolin, proanthocyanidin A2, 4‐methyl‐umbelliferyl‐N‐acetyl‐chitobiose, and (R)‐methysticin), organic oxygen compounds (3 DCCs, sesamose, A‐L‐arabinofuranosyl‐(1‐ > 2)‐[a‐D‐mannopyranosyl‐(1‐ > 6)]‐D‐mannose, and kanamycin), benzenoids (3 DCCs, 4‐Hydroxybenzyl alcohol, aniracetam, and emodin), 1ignans, neolignans, and regulated compounds (1 DCCs, hemsleyanoside), organic acids and derivatives (1 DCCs, phosphoramidon), and others (3 DCCs, lecanoric acid, physcion, and 4‐acetyl‐3‐hydroxy‐5‐methylphenyl beta‐D‐glucopyranoside). 4 DCCs downregulated DCCs (sesamose, necatorine, lecanoric acis, and (+)‐taxifolin) were important DCCs with the value of FC less than 0.5.

Similarly, a total of 60 DCCs were detected between DP3 and DP2, including 22 upregulated DCCs and 38 downregulated DCCs (Figure 5B). The upregulated DCCs included lipids and lipid‐like molecules (9 DCCs, e.g., 16‐oxoestrone, thromboxane B2, 15‐deoxy‐delta‐12,14‐PGJ2, physalolactone C, pentadeca‐7,10,13‐trienedioylcarnitine, (2‐acetyloxy‐3‐octadecoxypropyl)2‐(trimethylazaniumyl)ethyl phosphate, etc.), organic acids and derivatives (3 DCCs, zofenopril, bioadykinin fragment 1–5, and aspartame), phenylpropanoids and polyketides (3 DCCs, coumachlor, fukinolic acid, and 6‐hydroxyluteolin 6‐xyloside), alkaloids and derivatives (2 DCCs, 17‐*O*‐deacetylvindoline, and 7Z,14Z‐eicosadienoic acid), nucleosides, nucleotides, and analogs (2 DCCs, DCMP, and Dibutyryl cyclic gmp), benzenoids (1 DCC, aminosalicylic acid), and others (2 DCCs, SM(d14:0/2:0), and MG(TXB2/0:0/0:0)). Meanwhile, zofenopril, 16‐oxoestrone, SM(d14:0/2:0), and bioadykinin fragment 1–5 were important up DCCs with the value of FC greater than 2.0. The downregulated DCCs included organic acids and derivatives (8 DCCs, e.g., majoroside F6, mimosine, S‐lactoylglutathione, deferoxamine, N‐caffeoyltryptophan, etc.) phenylpropanoids and polyketides (7 DCCs, e.g., cyanidin 3‐rutinoside, limocitrin 3‐rhamnoside, isopeonidin 3‐sambubioside, biochanin A 7‐(6‐malonylglucoside), and kaempferol 3‐[6″‐(3‐hydroxy‐3‐methylglutaryl)glucoside]‐7‐glucoside, etc.), organoheterocyclic compounds (5 DCCs, mangiferin, angiferin, citrusinine I, stercobilin, nipradilol), alkaloids and derivatives (4 DCCs, camptothecin sodium, 4‐desacetylvinblastine hydrazide, 10‐hydroxycamptothecin, and lycorine), organic oxygen compounds (3 DCCs, acetyl CoA, chlorogenoquinone, and amygdalin), lipids and lipid‐like molecules (3 DCCs, PA(i‐21:0/8:0), quercilicoside A, (1R,4Ar,5S,7R)‐7‐methyl‐1‐[(2R,3S,4R,5R,6S)‐3,4,5‐trihydroxy‐6‐(hydroxymethyl)oxan‐2‐yl]oxy‐1,5,6,7a‐tetrahydrocyclopenta[c]pyran‐4a,5,7‐triol), benzenoids (2 DCCs, 14‐methoxymetopon, and N‐(6‐aminopyridin‐2‐yl)‐4′‐cyanobiphenyl‐4‐sulfonamide), lignans, lipids, and lipid‐like molecules (1 DCCs, manassantin B), nucleosides, nucleotides, and analogs (1 DCCs, 3′,5′‐Cyclic GMP), and others (4 DCCs, PI(20:4(6Z,8E,10E,14Z)‐2OH(5S,12R)/20:0), PI(18:1(11Z)/PGJ2), PS(6 keto‐PGF1alpha/18:3(9Z,12Z,15Z)), and PS(6 keto‐PGF1alpha/20:5(5Z,8Z,11Z,14Z,17Z))). Meanwhile, 8 DCCs (Na‐*p*‐hydroxycoumaroyltryptophan, 2″,6″‐diacetylorientin, glyceollin II, lycorine, PI(20:4(6Z,8E,10E,14Z)‐2OH(5S,12R)/20:0), amygdalin, biochanin A 7‐(6‐malonylglucoside), and 10‐hydroxycamptothecin) were important downregulated DCCs with the value of FC less than 0.4.

Only 35 DCCs were detected between DP4 and DP3 (Figure 5C), including 28 upregulated DCCs and 7 downregulated DCCs. The upregulated DCCs included lipids and lipid‐like molecules (6 DCCs, e.g., quercilicoside A, deoxycholic acid, medicoside G, (3b,16a,20R)‐3,16,20,22,25‐Pentahydroxy‐5‐cucurbiten‐11‐one3‐[glucosyl‐(1‐ > 6)‐glucoside], PA(i‐21:0/8:0), etc.), organoheterocyclic compounds (5 DCCs, nipradilol, citrusinine I, 6‐dehydrotestosterone glucuronide, stercobilin, ivabradine), phenylpropanoids and polyketides lipids and lipid‐like molecules (4 DCCs, 2″,6″‐diacetylorientin, biochanin A 7‐(6‐malonylglucoside), glyceollin II, and alpha‐solanine), organic acids and derivatives (4 DCCs, Na‐*p*‐hydroxycoumaroyltryptophan, deferoxamine, N‐caffeoyltryptophan, N‐octanoyl‐L‐homoserine lactone), alkaloids and derivatives (4 DCCs, lycorine, 10‐hydroxycamptothecin, 4‐desacetylvinblastine hydrazide, and camptothecin sodium), benzenoids (1 DCCs, *P*‐hydroxyfelbamate), organic 1,3‐dipolar compounds (1 DCCs, 2‐(2‐Nitroimidazol‐1‐yl)‐n‐(3,3,3‐trifluoropropyl)acetamide), and others (3 DCCs, PI(20:4(6Z,8E,10E,14Z)‐2OH(5S,12R)/20:0), PI(18:1(11Z)/PGJ2), and PS(6 keto‐PGF1alpha/18:3(9Z,12Z,15Z))). Meanwhile, Na‐*p*‐hydroxycoumaroyltryptophan, *P*‐hydroxyfelbamate, lycorine, PI(18:1(11Z)/PGJ2), PA(i‐21:0/8:0), 2″,6″‐diacetylorientin, PI(20:4(6Z,8E,10E,14Z)‐2OH(5S,12R)/20:0), 10‐hydroxycamptothecin, and ivabradine were important upregulated DCCs with the value of FC over than 2.0. The downregulated DCCs included lipids and lipid‐like molecules (2 DCCs, (2‐acetyloxy‐3‐octadecoxypropyl) 2‐(trimethylazaniumyl)ethyl phosphate, and 3‐methylthiopropionic acid), organic acids and derivatives (2 DCCs, hippuryl‐L‐lysine, and thiomorpholine 3‐carboxylate), phenylpropanoids and polyketides (1 DCC, coumachlor), organoheterocyclic compounds (1 DCC, porphyrinogen), and alkaloids and derivatives (1 DCC, delimotecan). Meanwhile, 3‐methylthiopropionic acid, delimotecan, and thiomorpholine 3‐carboxylate were important downregulated DCCs with the value of FC less than 0.5.

129 DCCs were detected between DP4 and DP1, including 91 upregulated DCCs and 38 downregulated DCCs (Figure 5D). The upregulated DCCs included lipids and lipid‐like molecules (26 DCCs, e.g., 16‐oxoestrone, isopropylmaleic acid, pentadeca‐7,10,13‐trienedioylcarnitine, 16‐ketoestradiol, plumieride, etc.), organic acids and derivatives (15 DCCs, e.g., cysteinyl‐alanine, ciraparantag, N‐eicosapentaenoyl aspartic acid, 2‐amino‐5‐phosphonopentanoic acid, asparaginylglutamine, etc.), organic oxygen compounds (15 DCCs, e.g., 2‐fucosyllactose, lichenin, cis‐5‐caffeoylquinic acid, cichorioside K, verbasoside, etc.), organoheterocyclic compounds (9 DCCs, decoquinate, stercobilin, loliolide, 4‐*O*‐(indole‐3‐acetyl)‐D‐glucopyranose, ivabradine, etc.), phenylpropanoids and polyketides (7 DCCs, e.g., luteolin 7‐glucuronide, kaempferol 3‐(6‐[4‐glucosyl‐p‐coumaryl]glucosyl)(1‐ > 2)‐rhamnoside, 2″,6″‐diacetylorientin, daunorubicin, nodakenetic, etc.), alkaloids and derivatives (6 DCCs, e.g., lycorine, 10‐hydroxycamptothecin, 17‐*O*‐deacetylvindoline, 4‐desacetylvinblastine hydrazide, camptothecin sodium, etc.), benzenoids (5 DCCs, 4‐benzyl‐2‐methyl‐1,2,4‐thiadiazolidine‐3,5‐dione, benzyl formate, 4‐vinylphenol, *p*‐hydroxyfelbamate, 4R,5R,6S‐trihydroxy‐2‐hydroxymethyl‐2‐cyclohexen‐1‐one 6‐(2‐hydroxy‐6‐methylbenzoate)), and others (8 DCCs, e.g., SM(d14:0/2:0), scorodocarpine A, N‐benzyloxycarbonylglycine, N‐[2‐(5‐Hydroxy‐1H‐Indol‐3‐yl)ethyl]icosanamide, PI(20:4(6Z,8E,10E,14Z)‐2OH(5S,12R)/20:0), etc.). Among them, 16‐oxoestrone, cysteinyl‐alanine, 4‐benzyl‐2‐methyl‐1,2,4‐thiadiazolidine‐3,5‐dione, 2‐fucosyllactose, *p*‐hydroxyfelbamate, Na‐*p*‐hydroxycoumaroyltryptophan, luteolin 7‐glucuronide, lichenin, nodakenetic, cis‐5‐caffeoylquinic acid, and lycorine were significantly upregulated DCCs with a value of FC over 3.0. While 38 downregulated DCCs included lipids and lipid‐like molecules (9 DCCs, e.g., glyceryl Monolinoleate, 18‐hydroxyoleate, lysoPA(18:2(9Z,12Z)/0:0), diosbulbinoside F, capsoside A, etc.), phenylpropanoids and polyketides (7 DCCs, e.g., dalbergioidin, proanthocyanidin A2, 4‐methyl‐umbelliferyl‐N‐acetyl‐chitobiose, lappaol, (+)‐taxifolin, etc.), organic acids and derivatives (6 DCCs, e.g., fuzlocillin, phosphoramidon, methionine sulfoxide, N(omega)‐hydroxyarginine, 2‐n‐propyl‐4‐oxopentanoic acid, etc.), benzenoids (5 DCCs, aniracetam, 3,4‐dihydroxyphenylacetic acid, emodin, 4‐hydroxybenzyl alcohol, and demethylzeylasteral), organic oxygen compounds (2 DCCs, sesamose and A‐L‐arabinofuranosyl‐(1‐ > 2)‐[a‐D‐mannopyranosyl‐(1‐ > 6)]‐D‐mannose), organic nitrogen compounds (1 DCC, histamine), nucleosides, nucleotides, and analogs (1 DCCs, 3′,5′‐cyclic GMP), lignans, neolignans, and regulated compounds (1 DCC, hemsleyanoside), and others (6 DCCs, e.g., fraxetin, 1‐palmitoyl lysophosphatidic acid, DG(5‐iso PGF2VI/0:0/2:0), physcion, lecanoric acid, etc.). Meanwhile, 7 DCCs (physcion, 3‐methylthiopropionic acid, lecanoric acid, (+)‐taxifolin, 3′,5′‐cyclic GMP, thiomorpholine 3‐carboxylate, and sesamose) were important DCMs with FC less than 0.5.

**FIGURE 6:**
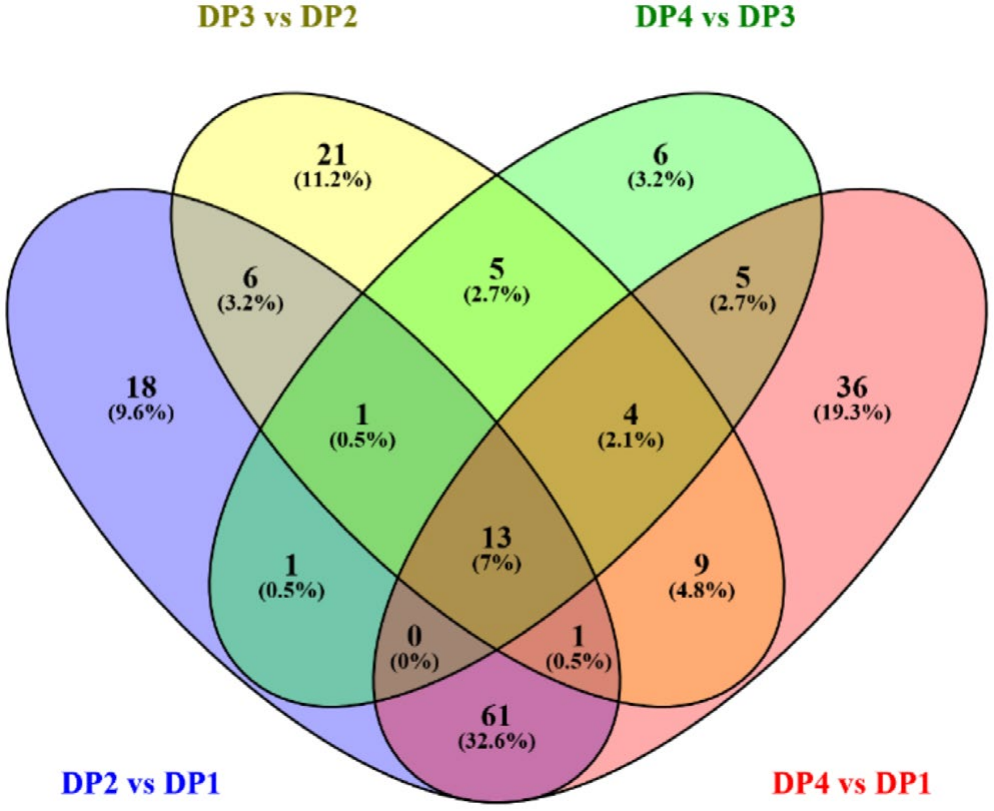
The Venny map of the number of DCCs during the dry processing in *C. arabica* from Yunnan province, China. 18, 21, 6, and 36 DCCs were unique in DP2 versus DP1, DP3 versus DP2, DP4 versus DP3, and DP4 versus DP1, respectively. 21 common DCCs existed between DP2 versus DP1 and DP3 versus DP2, and 23 common DCCs existed between DP4 versus DP3 and DP3 versus DP2. 13 DCCs were common in all comparative groups.

Figure 6 is a Venny map that shows the number of DCCs that are either shared or unique during the dry processing. The Venny map shows that 18, 21, 6, and 36 DCCs were unique to DP2 versus DP1, DP3 versus DP2, DP4 versus DP3, and DP4 versus DP1, respectively. Besides, compared to DCCs in DP4 versus DP3, the DCCs in DP2 versus DP1 and DP3 versus DP2 were richer. The number of DCCs decreased as processing times increased during the dry processing. In addition, common DCCs between DP2 versus DP1 and DP3 versus DP2 were found to be 21. 23 common DCCs were found between DP3 versus DP2 and DP4 versus DP3. 13 DCCs were common in all comparative groups. They were citrusinine I, PA(i‐21:0/8:0), lycorine, 2″,6″‐diacetylorientin, Na‐*p*‐hydroxycoumaroyltryptophan, stercobilin, 10‐hydroxycamptothecin, ivabradine, nipradilol, camptothecin sodium, deferoxamine, biochanin A 7‐(6‐malonylglucoside), and PI(20:4(6Z,8E,10E,14Z)‐2OH(5S,12R)/20:0).

## 4 Discussion

From out layer to bean, coffee beans are surrounded by silver skin, parchment, mucilage, pulp, and skin. Microorganisms play an important function in coffee fermentation before becoming green coffee beans by decomposing pectin (Lee et al. 2015). The microbial community structure was significantly influenced by the primary processing of coffee. Different primary processing methods also showed different microorganisms' characteristics. *C. arabica* using washed processing showed bacterial genera *Achromobacter*, *Tatumella*, *Weissella*, *Streptococcus*, and *Trichocoleus*, and fungial genera *Cystofilobasidium*, *Hanseniaspora*, *Lachancea*, *Wickerhamomyces*, and *Aspergillus* were predominant microorganisms (Shen et al. 2023b). *C. arabica* using semidry processing showed *Bacillus subtilis* , *Escherichia coli* , *Enterobacter agglomerans* , *Bacillus cereus* , and *Klebsiella pneumoniae* were the predominant bacteria, and *Pichia anomala*, *Torulaspora delbrueckii*, and *Rhodotorula mucilaginosa* were the dominant yeasts (Vilela et al. 2010). Furthermore, the microbial community structure also was significantly influenced in different regions. For example, in Colombia, *Leuconostoc* and *Acetobacter* were predominant genera (Cruz‐O'Byrne et al. 2021). In Korea, the producing polygalacturonase species, *Wickerhamomyces anomalus* and *Saccharomycopsis fibuligera*, and *Papiliotrema flavescens* were predominant species. *S. cerevisiae* was also found, which had a high potential in producing pectin methylesterase (Haile and Kang 2019). In addition, coffee from different surroundings also showed significant disintegration. *Leuconostoc* and *Weissella* were the most common lactic acid bacteria at an altitude of 800 m, whereas it was *Lactococcus lactis subsp. lactis* at 1200 m (Leong et al. 2014).

Lipids, acids, polysaccharides, caffeine, chlorogenic acids, trigonelline, and other components are important to coffee flavor (Sunarharum et al. 2014). For example, lipids, as the main chemical compound in coffee, reached 12.88–16.29 g/100 g in green coffee beans (Zhu et al. 2022). Among them, C24:0, C22:0, C20:0, C18:0, C17:0, and C16:0 were determined as discriminatory compounds for different geographical origins of green coffee beans (Zhu et al. 2021). Moreover, lipids could change and migrate to the surface of roasted coffee beans by roasting. Then, they were extracted into the coffee brew to contribute to the perceived texture and mouthfeel of the coffee brew (Sunarharum et al. 2014). Sugar is an important coffee flavor compound in coffee, which often is affected by postharvest treatment and roasting degree (Knopp et al. 2006; Freitas, Borges, Castro, et al. 2024; Freitas, Borges, Vidigal, et al. 2024). Sugars, such as sucrose, D‐galactose, pentose, D‐fructose, D‐galactose, stachyose, and sesamose, were found in dry processing. The contents of fructose, glucose, galactose, arabinose, and mannose in dry processing were higher than in wet processing (Knopp et al. 2006). They remained unchanged or increased in dry processing while decreasing in wet processing (Sunarharum et al. 2014). However, sucrose was not affected by processing (Knopp et al. 2006). Moreover, the amino acid is also very important, which can form coffee flavor compounds with sugar by Maillard reaction. *γ*‐aminobutyric acid in dry processing also was higher than in wet processing (Bytof et al. 2005). The analysis of the characteristic compounds in different primary processings showed that fructose and γ‐aminobutyric acid were the characteristic compounds in dry processing, chlorogenic acids (especially caffeoyl quinic acids), malic acid, and sucrose in the wet processing. While chemical compounds in the semidry processing were the intermediary metabolite between dry and wet processing (Happyana et al. 2024). The primary processing not only influences the precursor compounds of green coffee beans but also influences the coffee flavors and characteristics (Zhai et al. 2024) because of the function of microorganisms (Shen et al. 2023b; Cruz‐O'Byrne et al. 2021). For example, 3‐deazaadenosine had a strong positive correlation with *Burkholderia*, *Dyella*, *Wickerhamomyces*, and *Candida*, whereas a strong negative correlation with *Tatumella* (Shen et al. 2023b) in coffee primary processing. 4‐Hydroxy‐6‐methyl‐3‐(1‐oxobutyl)‐2H‐pyran‐2‐one displayed a strong positive correlation with *Burkholderia*, *Dyella*, and *Candida*. (PC)14:1(9Z)/20:19(11Z), PC(18:2(9Z,12Z)/18:1(11Z)), and PE‐NMe(22:2(13Z,16Z)/16:1(9Z)) showed a strong negative correlation with *Hanseniaspora* (Shen et al. 2023b). *Lactobacillales*, *Pichia* , and Pseudomonas positively correlated with the cup quality of coffee.

The number of DCCs decreased with the dry processing development. In DP2 versus DP1, the number of DCC was the richest, followed by DP3 versus DP2. This changed trend may relate to the loss of water and decrease of enzymes during dry processing. Moreover, 5‐caffeoylquinic acid was the dominant compound in green coffee beans (Ali et al. 2022) and an important changed compound during the dry processing. Sucrose, glucose, and fructose as important precursor compounds for coffee flavor, the change was not significant. In a comparison with the related increase of 2‐fucosyllactose and 3‐fucosyllactose in DP2 versus DP1, sesamose was decreased. Cysteinyl‐alanine, N‐eicosapentaenoyl aspartic acid, N‐acetyl‐7‐*O*‐acetylneuraminic acid, and N‐benzyloxycarbonylglycine were significantly increased in DP2 versus DP1.

Therefore, during the primary processing, the chemical compounds in coffee were changed along with the change of microorganisms during primary processing, which would influence the coffee flavor finally.

## 5 Conclusions

The microbial community structure and chemical compounds of *C. arabica* from Yunnan province in the coffee fermentation process using the dry process were analyzed and compared. 25 phyla and 31 genera for bacteria were found, in which *Tatumella*, *Klebsiella*, *Gluconobacter, Brevundimonas*, *Staphylococcus*, *Pantoea*, *Bacillus*, and *Leuconostoc* were the predominant genera. As well, 5 phyla and 31 genera for fungi were found, in which *Candida*, *Lachancea*, *Aschersonia, Cercrospora*, *Pichia*, *Cladosporium*, *Hanseniaspora*, *Hannaella*, *Papiliotema*, and *Colletotrichum* were the top ten predominant fungi genera. Moreover, a total of 1551 chemical compounds from 15 superclasses were identified during the dry processing. Furthermore, based on the analysis of differentially changed chemical compounds, 101 DCCs were detected in DP2 versus DP1, including 80 upregulated and 21 downregulated DCCs. In DP3 versus DP2, 60 DCCs were detected, of which 22 were upregulated and 38 were downregulated DCCs. In DP4 versus DP3, only 35 DCCs were detected, of which 28 were upregulated and 7 were downregulated DCCs. Overall, 129 DCCs were identified in DP4 versus DP1, with 91 upregulated and 38 downregulated DCCs. At the beginning of the dry processing, the chemical compounds of coffee quickly changed. With the processing development, the change of chemical compounds was stabilizing.

## Author Contributions

**Xiaojing Shen:** data curation (equal), formal analysis (equal), funding acquisition (equal), writing – original draft (equal). **Qi Wang:** data curation (equal), formal analysis (equal). **Biao Yuan:** data curation (equal), formal analysis (equal). **Zhiheng Yin:** resources (equal), software (equal). **Muzi Li:** investigation (equal), visualization (equal). **Runxin Shi:** investigation (equal), visualization (equal). **Kunyi Liu:** conceptualization (equal), data curation (equal), formal analysis (equal), investigation (equal), methodology (equal), software (equal), writing – review and editing (equal). **Wenjuan Yuan:** conceptualization (equal), data curation (equal), formal analysis (equal), investigation (equal), methodology (equal), software (equal), writing – review and editing (equal).

## Conflicts of Interest

The authors declare no conflicts of interest.

## Data Availability

Data available on request from the authors. The data that support the findings of this study are available from the corresponding author upon reasonable request.
